# Alcohol intake, smoking, self-medication practices and burden of anaemia among traders in Tamale metropolis of Ghana

**DOI:** 10.1186/s13104-023-06480-2

**Published:** 2023-09-12

**Authors:** Nsoh Godwin Anabire, George Doopaar Billak, Gideon Kofi Helegbe

**Affiliations:** 1 Department of Biochemistry and Molecular Medicine, School of Medicine, University for Development Studies, Tamale, Ghana; 2 West African Centre for Cell Biology of Infectious Pathogens (WACCBIP), Department of Biochemistry Cell and Molecular Biology, University of Ghana, Ghana, Accra Ghana

**Keywords:** Anaemia, Haemoglobin, Smoking, Alcohol, Traders, Market women, Smoking, Self-medication

## Abstract

**Objective:**

Lifestyle choices including physical inactivity, smoking, abuse of alcohol and drugs, unhealthy diet are common among traders and market women and these behavioural activities predispose individuals to ill-health conditions including cardiovascular diseases and chronic anaemia. We evaluated lifestyle choices such as alcohol intake, smoking and resorting to self-medication among traders in the Tamale Central market in Ghana. We then associated these lifestyle choices with anaemia.

**Results:**

A total of 400 participants were recruited for this study. Haemoglobin (Hb) levels of participants were measured using Mission® Plus Hb meter and anaemia was diagnosed by Hb < 12 g/dl for non-pregnant females and Hb < 13 g/dl for males. Of the participants, a majority (69.3%) were males, and most of them (56.0%) were within 18–35 years age bracket. While alcohol intake and smoking were uncommon, self-medication was a common practice among the participants. Anaemia was a common condition; diagnosed in 44.5% of participants, but was independent of age, alcohol intake and smoking. However, anaemia was more common in females *(χ*^*2*^ *= 15.9, p < 0.001)* and was associated with self-medication *(χ*^*2*^ *= 5.7, p = 0.017)*. We recommend that traders in the Tamale metropolis should seek routine health check-ups to help avert adverse health consequences associated with anaemia.

## Introduction

Lifestyle choices are actions that can have a beneficial or negative impact on the human body [1]. It is recognized that some behaviours, such as smoking, drinking alcohol harmfully, abusing drugs, being physically inactive, and eating unhealthily, among others, predispose people to health issues like cardiovascular illnesses and chronic anaemia [2]. Retaining optimal physical activities and body weight, adhering to a healthy diet, non-smoking and limiting alcohol consumption are some lifestyle activities that are widely known to promote good health [3, 4]. According to statistics from the World Health Organization (WHO), excessive salt intake, smoking (mostly tobacco), excessive alcohol consumption, and physical inactivity are each responsible for 1.7 million, 6.0 million, 3.3 million and 3.2 million annual deaths, respectively [5]. The combination of lifestyle choices (drinking excessive amounts of alcohol, smoking, eating poorly, and not exercising) is also reported to have a significant impact on overall and cause-specific death [6].

Anaemia is a condition that occurs when there are insufficient red blood cells in the blood to properly oxygenate the body tissues. Usually, this is accompanied by decreased haemoglobin levels. The WHO defines anaemia as having a haemoglobin level of less than 12 g/dL in non-pregnant women and less than 13 g/dL in men [7]. Research have demonstrated that haemoglobin concentration is the most accurate predictor of anaemia at the population level, even though it alone cannot identify the underlying cause of anaemia [8]. Anaemia as a global public health problem affects low-, middle- and high-income countries at different degrees and its prevalence varies with socio-economic status [9].

Population-based studies have documented that life styles choices such as heavy cigarette smoking and alcohol consumption are found to be correlated with iron metabolism and seen as risk factors of anaemia [10–12]. Despite these studies, there is little to no knowledge on how lifestyle choices relate with anaemia in a predominantly domicile population of market women and traders. In our prior study [13], we had reported lifestyle determinants among predominantly domicile population of traders in the Tamale metropolis of Ghana. Using the same population, in this current study, we evaluated the association between the lifestyle choices (smoking, alcohol consumption and self-medication) and anaemia outcomes.

## Materials and methods

This research was a cross-sectional study that took place at the Tamale Central Market involving traders [13]. All the study participants signed a consent form and allowed for their information to be used for the purpose of this research work without personal identification. A total of 400 study participants (males and females) aged 18 years and above were recruited for this study. Structured questionnaires were used to obtain their demographic information and lifestyle choices and other relevant information that was crucial for the study [13]. The demographic and lifestyle characteristics included age, gender, alcohol intake, smoking and self-medication [13]. The skin of the fingers of the study participants were pricked with the help of lancets and their haemoglobin levels were measured the using Mission® Plus Hb meter. Anaemia was then diagnosed by Hb < 12 g/dL for non-pregnant females and Hb < 13 g/dL for males as defined by the WHO. Demographic and lifestyle variables were then used for association analysis with anaemia. This study was approved by the joint institutional review board of the School of Medicine and Health Sciences (SMHS) and the School of Allied Health Sciences (SAHS) of the University for Development Studies, Tamale, Ghana. Recruitments of participants and all methods carried on the participants and blood samples were done in accordance to the approved protocol by the joint institutional review boards, which follows the Helsinki Declaration. All methods were carried out in accordance with the guidelines and regulations laid down by the ethics committee. Participants in the study were given confidentiality assurances and were made aware that their participation was voluntary and that they could leave the study at any time without facing any repercussions. Data analysis was done using SPSS version 26. Association of anaemia with demographic factors and lifestyle choices was evaluated using chi-square or fisher’s exact tests. A *P*-value $<$0.05 was considered statistically significant.

## Results

### Background characteristics of participants

The general characteristics of the study participants are shown in Table 1. The socio-demographic and lifestyle choices data have previously been reported in our prior study [13]. With regards to sex, 69.3% (n = 277) were males while 30.7% were females (n = 123). The mean age ± SD was 37.1 ± 13.1 years. According to age distribution, 56% (n = 224) of the participants were within the 18–35 years age bracket, 31.8% (n = 127) were within the of 36–55 years age bracket while 12.2% (n = 49) were within 55 + years age bracket. The practice of self-medication was seen to dominate among the participants with 50.5% (n = 202) of them practicing self-medication. Mainly antimalarials and painkillers were used as self-medication whenever the participants felt sick following signs such as chest and bodily pains. Alcohol intake was low among the participants, with 3.0% (n = 12) of them practicing it while the other 97.0% (n = 388) did not indulge in alcohol consumption. Similarly, cigarette smoking was low among the participants; 94.3% (n = 377) of the participants did not engage in the activity of smoking while 5.7% (n = 23) were seen to be engaging in such activity. The mean Hb ± SD was 12.8 ± 1.7 g/dL (range: 5.5–17.2 g/dL). A total of 44.5% (n = 178) of the study participants had anaemia while the remaining 55.5% (n = 222) participants had no anaemia (Table 1).

**Table 1:** Distribution of demographic, lifestyle and anaemia statuses among study participants

Factor	Category	n (%)	95% CI
Sex	Male	277 (69.3)	64.5–73.7
	Female	123 (30.8)	26.3–35.5
Age (years)	18–35	224 (56.0)	51.0–60.9
	36–55	127 (31.8)	27.2–36.6
	55+	49 (12.3)	9.2–15.9
Self-medication	Yes	202 (50.5)	44.5–55.5
	No	198 (49.5)	44.5–54.5
Alcohol intake	Yes	12 (3.0)	1.6–5.2
	No	388 (97.0)	94.8–98.4
Smoking	Yes	23 (5.8)	3.7–8.5
	No	377 (94.3)	91.5–96.3
Anaemia	Yes	178 (44.5)	39.6–49.5
	No	222 (55.5)	50.5–60.4

95% CI: 95% confidence interval

### Association of anaemia with demographic and lifestyle choices

The association between the sex of the participants and anaemia is shown in Table 2. It can be seen that out of the 277 (100.0%) males, 37.9% (n = 105) were anaemic while 62.1% (n = 172) were not anaemic. Also, out of the 123 (100.0%) females, 59.3% (n = 73) were anaemic while the remaining 40.7 (n = 50) were not. The statistics suggest that the prevalence of anaemia was higher in females as compared to the males (χ^2^ = 15.9, p < 0.001).

The association between age of the participants and anaemia is shown in Table 2. Across the different age groups, the prevalence of anaemia was higher (59.2%) in participants within the 55 + age bracket, followed by those (44.9%) within the 36–55 years age bracket, and those (41.1%) within the 18–35 age group. That notwithstanding, age and anaemia did not show any significant association, as the proportions of participants with anaemia were similar across the different age groups (χ^2^ = 5.35, p = 0.069). This means that among the participants, anaemia outcome was independent of age.

The association between self-medication and anaemia is shown in Table 2. Of the participants (n = 202) that resorted to self-medications, 38.6% of them were anaemic while 61.4% were not anaemic. Of the participants (n = 198) who were not involved in self-medication, 50.5% were anaemic while 49.5% were not anaemic. There was a significant association between self-medication and anaemia, as the proportion of participants that resorted to self-medication had lower rates of anaemia compared to those who did not (χ^2^ = 5.7, p = 0.017).

The association between alcohol intake and anaemia is shown in Table 2. A total of 12 participants were involved in alcohol intake, out of which 16.7% were anaemic while 83.3% were not anaemic. Of the remaining 388 participants who were not engage in alcohol intake, 45.4% were anaemic while 54.6% were not anaemic. No significant association was found between alcohol consumption and anaemia; the rates of anaemia were similar between participants who consumed alcohol and those who did not (χ^2^ = 3.88, p = 0.074).

The association between smoking and anaemia among the study participants is shown in Table 2. Of the 23 participants who engaged in smoking, 26.1% were anaemic while 73.9% were not anaemic. Of the 377 participants that were not involved in any form of smoking, 45.6% were anaemic while 54.4% were not. No significant association was found between smoking and anaemia; the rates of anaemia were similar between participants who smoked and those who did not (χ^2^ = 3.35, p = 0.067).

**Table 2:** Chi-square analysis of association of anaemia with demographic factors and lifestyle choices

Factor	Category	Anaemia		(χ^2^), *P-value*
		Yes, n (%)	No, n (%)	
Sex	Male	105 (37.9)	172 (62.1)	15.86 < 0.001
	Female	73 (59.3)	50 (40.7)	
Age (years)	18–35	92 (41.1)	132 (58.9)	5.35 0.069
	36–55	57 (44.9)	70 (55.1)	
	55+	29 (59.2)	20 (40.8)	
Self-medication	Yes	78 (38.6)	124 (61.4)	5.72 0.017
	No	100 (50.5)	98 (49.5)	
Alcohol intake	Yes	2 (16.7)	10 (83.3)	3.88 0.074
	No	176 (45.4)	212 (54.6)	
Smoking	Yes	6 (26.1)	17 (73.9)	3.35 0.067
	No	172 (45.6)	205 (54.4)	

*χ*^*2*^: *Chi-square value, analysed using Chi-square or Fisher’s exact test. P-value considered statistically significant at < 0.05 (2- tailed).*

## Discussion

Overall, the prevalence of anaemia among traders in the Tamale Central Market was 44.5% [13]. This prevalence, however, was more common in females than in males and this is consistent with findings in Sub-Saharan Africa that women (especially those in the reproductive age) have a significantly higher prevalence of anaemia than men [14–16]. This observation can be attributed to a number of factors including low education and wealth index [14], iron deficiency and parasitic diseases and infections [17], among females in Sub-Saharan Africa.

Alcohol intake, smoking and self-medication are the lifestyle choices that were evaluated among the traders and were subsequently associated with anaemia. It has been documented that chronic ingestion of alcohol alters the hematopoietic system resulting in folate deficiency [18, 19]. Also, reports indicates that alcohol intake increases the risk of anaemia and correlates with reduced levels of Hb and RBCs [19, 20]. In this current study, however, alcohol consumption was found to be independent of anaemia, and this observation may be due to the low incidence of alcoholism as observed among the study participants. Predominantly, most residents of Tamale (the study area) are Muslims, and the low incidence of alcoholism could be attributed to the Islamic practice that forbids alcohol consumption.

Cigarette smoking is known to promote macrocytosis through changing the amounts of folic acid and vitamin B12 [21, 22]. It lowers vitamin C levels, which lowers iron absorption and makes a person more likely to develop iron deficiency anaemia. Continuous cigarette smoking can have serious negative effects on haematological parameters (including haemoglobin, haematocrit, and red blood cell count) in a healthy population, and these changes can raise the risk of atherosclerosis and cardiovascular diseases [23]. In that regard, a number of studies have shown that the levels of Haemoglobin are low in smokers [24, 25]. That notwithstanding, evidence suggests a protective role of cigarette smoking against anaemia due to erythropoietin-stimulating influence of smoking-induced increase in carbon monoxide [26, 27]. To this effect, studies have confirmed a negative correlation between cigarette smoking and the risk of anaemia [28–30]. In this current study, we observed cigarette smoking was not significantly associated with anaemia, probably due to the lower smoking incidences observed in the study participants. Tamale (the research area) has a large Muslim population, and the Islamic practice that restricts cigarette smoking may be responsible for the low incidence of cigarette smoking among the study participants.

Self-care refers to actions or behaviours people perform on their own behalf to maintain their health, fend off illness, and stay healthy. One aspect of self-care is self-medication. According to the WHO, self-medication refers to the use of drugs to treat self-diagnosed disorders, symptoms or the intermittent or combined use of prescribed drugs for chronic or recurrent disease or symptoms [31]. A major shortfall of self-medication is the lack of clinical evaluation of the condition by a trained medical professional which could result in missed diagnosis and delay in appropriate treatment [32]. The activity of self-medication was high among the study participants, and this is consistent with other reports in Ghana [33]. In this study, the most self-medicated drugs were antimalarials and painkillers and the number one reason that accounted for this activity was previous experience with symptoms. A recent study among inhabitants of Ghana revealed natives are quite knowledgeable about the causes, symptoms, and alternate preventative measures of malaria, and that before taking an antimalarial, locals sought medical advice or completed a rapid diagnostic test for malaria [34]. The aforementioned findings may account for the significant lower prevalence of anaemia among participants that resorted to self-medication considering malaria is a prevalent condition in the study area [35, 36].

This study could not account for the varying degrees of the lifestyles choices such as chronicity of smoking and alcohol consumption, and the quantity of alcohol or cigarettes consumed per day. These data if collected could have been helpful in linking these lifestyle choices with anaemia. It is quite encouraging that alcohol consumption and cigarette smoking were rarely practiced among the traders in Tamale Metropolis of Ghana. That notwithstanding a larger study will be helpful in substantiating the findings of our study. Further studies are also needed to understand the link of self-medication practices with malaria disease severity, antimalarial drug resistance patterns and other infectious diseases including COVID-19.

## Conclusion

Anaemia was a common condition diagnosed in traders in the Tamale metropolis of Ghana, but was independent of age, alcohol intake and smoking. However, anaemia was more common in females and was associated with self-medication. We recommend traders in Tamale metropolis should seek routine health check-ups to help avert adverse health consequences associated with anaemia.

## Data Availability

The datasets supporting the findings of this article are available in this manuscript.

## Abbreviations

Hb: haemoglobin
WHO: World Health Organisation
RBCs: Red blood cells
